# Blood stream infections in Stevens Johnson Syndrome and Toxic Epidermal Necrolysis: Risk factors and association with poor outcome

**DOI:** 10.12669/pjms.40.11.9360

**Published:** 2024-12

**Authors:** Tazein Amber, Saadia Tabassum, Saad Bin Zafar Mahmood, Syed Ahsan Ali, Umair Javed, Muhammad Zain Mushtaq

**Affiliations:** 1 Tazein Amber, MBBS, FCPS, SCE(Derm)UK. Section of Dermatology, Department of Medicine, Aga Khan University Hospital, Karachi, Pakistan; 2 Saadia Tabassum, MBBS, FCPS. Section of Dermatology, Department of Medicine, Aga Khan University Hospital, Karachi, Pakistan; 3 Saad Bin Zafar Mahmood, MBBS, FCPS. MRCP Section of Internal Medicine, Department of Medicine, Aga Khan University Hospital, Karachi, Pakistan; 4 Syed Ahsan Ali, MBBS, FCPS. Section of Internal Medicine, Department of Medicine, Aga Khan University Hospital, Karachi, Pakistan; 5 Umair Javed, MBBS. Section of Internal Medicine, Department of Medicine, Aga Khan University Hospital, Karachi, Pakistan; 6 Muhammad Zain Mushtaq, MBBS, FCPS, MRCP. Section of Internal Medicine, Department of Medicine, Aga Khan University Hospital, Karachi, Pakistan

**Keywords:** Steven Johnsons Syndrome, Toxic Epidermal Necrolysis, Blood Stream Infections, Severe Cutaneous Adverse Reactions, Mortality

## Abstract

**Background & Objective:**

Blood Stream Infections (BSI) are considered a significant cause of morbidity and mortality in patients with Stevens Johnson Syndrome (SJS) and Toxic Epidermal Necrolysis (TEN). We aimed to identify risk factors for BSI upon admission, highlight clinical and microbiological findings and ascertain the frequency of mortality in patients with BSI in SJS/TEN

**Methods:**

A retrospective cross-sectional study over 12 years (2011-2022) was performed in the department of medicine at a tertiary care hospital in Pakistan. All patients admitted with the diagnosis of SJS or TEN were included: from the health information management system. We included clinical and microbiological details, reviewed medical charts, and filled out a predesigned proforma.

**Results:**

A total of 100 patients were admitted with SJS or TEN. The majority (55%) were of age greater than 40 years and had female preponderance (57%). Sixty five patients had a prior history of using a precipitating drug. BSI was seen in 19 patients; 68.4% had a mono-microbial infection, while 31.5% had a poly-microbial infection. In total, 10 organisms were identified, *Staphylococcus aureus* being the most common isolate followed by *Enterococcus*. Twelve patients required intensive care monitoring while 33 patients had hospital stays of equal or more than seven days. The overall mortality rate was 15% while it was 60% in those with BSI. SCORTEN score of ≥4 had a significant impact on mortality (60% deaths).

**Conclusion:**

Vigilant monitoring and early detection of BSI in SJS/TEN patients, especially those presenting with high SCORTEN scores can enhance clinical outcomes.

## INTRODUCTION

Stevens-Johnsons Syndrome (SJS) and Toxic Epidermal Necrolysis (TEN) are among the life-threatening dermatological emergencies that fall under Severe Cutaneous Adverse Reactions (SCARs). These are immune-mediated type-IV hypersensitivity response characterized by keratinocyte death and detachment from the epidermis, secondary to drug exposure or less commonly after an infection.^1^ The annual incidence of SJS in adults ranges from 3.9 to 5.3 per million globally, while 0.4 to 1.45 per million for TEN. Furthermore, reported mortality is 1-5% for SJS and 25-35% for TEN.^2^ Interestingly, a study concluded that SJS and TEN are two-fold more common in the Asian population when compared to Caucasians.^3^

Data from the Pakistani population showed an incidence of 1.89 per million per year.^1^ As already known, SJS and TEN in children are caused more commonly by preceding viral or bacterial infections, whereas, drugs are more common triggers of this syndrome in adults.^4^ Inciting drugs may belong to any class, ranging from anti-epileptics, and antipsychotics to antibiotics and Non-Steroidal Anti-Inflammatory Drugs (NSAIDs). Genetic predisposition has also been linked to the Asian population having the HLA-B1502 gene.^5^ The gold standard of treatment includes the withdrawal of the offending drug and supportive care.^6^

Blood Stream Infections (BSI) are considered a significant reason for morbidity and poor outcomes in patients with SJS and TEN with a mortality rate ranging from 20-50%.^6^ Moreover, Intensive Care Unit (ICU) admissions are more frequent in patients in whom hospital stay is complicated with BSI.^6^-^10^ Furthermore, the literature suggests that prior comorbidities like cardiovascular diseases, low hemoglobin, and total body surface area of more than 10% are associated with the development of bacteremia.^11^

The challenge while treating SJS/TEN is that on one hand guidelines suggest against the use of prophylactic antibiotics, as it may lead to the development of resistance and increased mortality, however on the other hand empiric antibiotic is often required when there is suspicion of sepsis.^10^,^12^,^13^ We aimed to identify risk factors for BSI upon admission, determine the epidemiology of BSI, highlight clinical findings and ascertain the frequency of mortality in patients with BSI in SJS and TEN patients;, thus aiding in the early detection of high-risk patients.

## METHODS

A retrospective, cross-sectional study was performed in the department of medicine at the Aga Khan University Hospital, which is a Joint Commission International Accredited (JCIA) tertiary care hospital in Karachi, Pakistan.

### Ethical Approval:

Institutional ethical review approval (ERC # 2019-1838-4646, dated August 21, 2019) was obtained for this non-interventional analysis of medical records before the start of the study.

**Fig.1:**
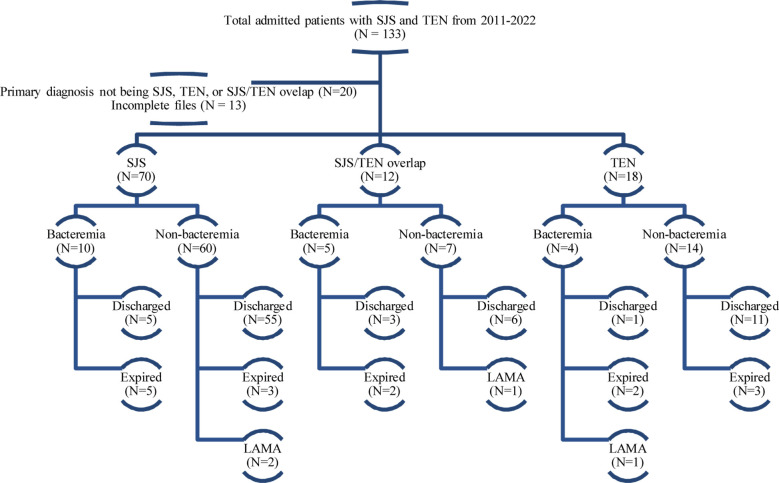
Diagrammatic flow of patient data extraction.

We reviewed 133 patients from the health information management system (HIMS) using International Classification of Diseases-9 (ICD-9) coding over 12 years (2011 till 2022). All patients admitted to the medicine ward with the primary diagnosis of SJS, TEN or SJS/TEN overlap to our center were included. The disease spectrum can be differentiated based on Total Body Surface Area (TBSA) and is termed as SJS with <10% involvement, whereas when it is >30% it is called TEN. In between lies an entity called SJS/TEN overlap syndrome. A total of 133 cases were extracted from HIMS, out of which 20 were excluded due to the primary diagnosis at the time of discharge not being SJS, TEN or SJS/TEN overlap. A further 13 patients were excluded because of incomplete files, leaving behind a total of 100 patients (Fig.1).

We included clinical and microbiological details from the hospital database, reviewed medical charts and filled predesigned proforma. Variables recorded at admission and during hospital stay included demographic details, comorbidities, clinical manifestations, SCORTEN score, inciting drug/s, microorganisms in blood cultures, length of hospital stay, ICU admission, complications (Acute Kidney Injury (AKI), troponin leak, derangement of liver enzymes or coagulation tests, electrolyte imbalance and respiratory failure) and outcome (discharge or death). SCORTEN score has been proposed for prognostication of SJS and TEN, which includes age, heart rate, and presence of malignancy, serum urea, bicarbonate, and glucose levels along with TBSA involvement. It is calculated at the time of admission, ideally within the first 48 hours of disease and precisely predicts mortality rate.

BSI was defined as when a blood culture showed growth of at least one pathogen, which was further classified into mono-microbial (one microorganism) and poly-microbial (two or more microorganisms). All organisms along with their antibiotic sensitivities were recorded. Clinical manifestations of BSI were divided into those having sepsis and septic shock. Sepsis was defined as a potentially life-threatening organ dysfunction due to dysregulated response to infection.^14^

Septic shock included patients who fulfill the criteria for sepsis but despite adequate fluid resuscitation, require vasopressors to maintain a mean arterial pressure (MAP) ≥65 mmHg and have a lactate >2 mmol/L (>18 mg/dL).^15^

Analyses were performed using SPSS software version 19. Continuous variables like age, SCORTEN score, and length of hospital stay were categorized and expressed as percentages and frequencies along with other categorical variables like gender, disease classification, medical history, spectrum of bloodstream infections, and outcome. Comparisons between bacteremia and non-bacteremia groups were made using χ2 or Fisher’s exact test for categorical data. We considered a two-sided *P-*value of <0.05 as statistically significant.

## RESULTS

The study comprised of 100 patients, of whom 70 (70%) were labeled as SJS, 18 (18%) as TEN and 12 (12%) were diagnosed with SJS/TEN overlap syndrome. The mean age was 41.5 ± 14.7 years, with 57 (57%) females. Majority (73%) patients presented with only sepsis at the time of admission. Hypertension (39%) was the most prevalent comorbidity while three patients had prior history of SJS. Unfortunately, those three patients were prescribed medications with the same generic name but different brand names, which led to the development of TEN during their subsequent admission. Baseline characteristics, past medical history, SCORTEN scores, precipitating cause, and outcomes of patients have been listed in Table-I. The mean SCORTEN score was 2.4 ± 1.3, with most patients having a score of less than 4 (76%). The majority (81%) of our patients were safely discharged. The majority (67%) of our patients had a history of medication use prior to the development of SJS/TEN (Table-II). Most of the cases in our study were found to be secondary to antibiotics (24%) followed by NSAIDs (17%).

**Table-I:** Comparison of baseline and clinical characteristics among bacteremia and non-bacteremia groups

Patient characteristics	Total (n=100)	Bacteremia (n=19)	Non-bacteremia (n=81)	P-value
**Age**				
Less than 40 years	45	9	36	0.818
Greater than 40 years	55	10	45	
**Gender**				
Female	57	8	35	0.930
Male	43	11	46	
**Disease classification**				
SJS	70	10	60	
TEN	18	4	14	0.077
SJS and TEN overlap	12	5	7	
**Past medical history**				
Hypertension	39	7	32	0.830
Diabetes	22	5	17	0.614
Neuro-psychiatric illness	17	4	13	0.601
Chronic Kidney Disease	10	4	6	0.074
Ischemic Heart Disease	9	4	5	0.041
Malignancy	4	2	2	0.162
Respiratory Disease	3	2	1	0.033
Autoimmune diseases	2	0	2	0.489
Endocrine diseases	1	0	1	0.626
Chronic liver diseases	1	0	1	0.626
Prior history of SJS	3	0	3	0.528
**Clinical manifestation**				
No sepsis	8	0	8	
Sepsis	73	9	64	<0.001
Septic Shock	19	10	9	
**Positive history of precipitating drug use**	67	14	53	0.378
**SCORTEN score**				0.040
Less than 4	76	11	65	
Equal to or more than 4	24	8	16	
ICU admission	12	7	5	<0.001
**Complications**				
Electrolyte imbalance	56	17	39	0.001
Acute kidney injury	43	11	32	0.145
Liver injury	40	12	28	0.026
Deranged coagulation	20	11	9	<0.001
Elevated troponin	13	6	7	0.007
**Infective parameters [Median (IQR)]**				
C-reactive protein (ug/mL)	12.7 (4.2-17.4)	13.7 (4.6-19.0)	12.0 (3.9-16.9)	0.411
Procalcitonin (ng/mL)	1.1 (0.2-6.4)	9.7 (0.9-35.7)	0.4 (0.1-2.8)	0.007
**Length of stay**				
Less than 7 days	67	12	55	
Equal or more than 7 days	33	7	26	0.692
**Outcome**				
Discharged	81	9	72	
Expired	15	9	6	<0.001
LAMA	4	1	3	

**Table-II:** Inciting Drugs

Drug	Frequency
Antibiotics	24
Penicillin	5
Cephalosporin	3
Azithromycin	7
Trimethoprim/Sulfamethoxazole	5
Fluoroquinolones	3
Metronidazole	1
Anti-inflammatory	19
NSAIDs	17
Steroids	2
Paracetamol	8
Antidepressants/Antipsychotics	5
Fluoxetine	2
Sertraline	1
Quetiapine	2
Antiepileptics	4
Lamotrigine	2
Carbamazepine	2
Herbal/Homeopathic	4
Allopurinol	3

BSI was seen in 19 out of 100 patients during hospital stay; 13 out of 19 (68.4%) had a mono-microbial infection, while 6 (31.5%) had a poly-microbial infection. In total, 10 organisms were identified, with *Staphylococcus aureus* (31.5%) being the most common isolate. Amongst those with poly-microbial infections, one patient had a combination of four microorganisms (*Staphylococcus aureus, E. coli, Acinetobacter*, and *Enterococcus*) in a single blood culture. The epidemiology of BSI is illustrated in Table-III.

**Table-III:** Micro-organisms

Blood Stream Infection Mono-microbial	19 13 (68.4%)
Poly-microbial	6 (31.5%)
***Micro-organisms isolated***	
**Bacteria**	**27**
**Gram Positive**	**17**
Staphylococcus aureus	6
MSSA	5
MRSA	1
Enterococcus	5
VSE	3
VRE	2
Staphylococcus epidermidis	2
Staphylococcus saprophyticus	2
Corynebacterium	2
**Gram Negative**	**10**
Escherichia coli	4
Acinetobacter spp.	4
Aeromonas	1
Burkholderia cepacia	1
**Fungi**	**4**
Candida spp.	4
***Polymicrobial combinations***	6
1.MSSA, Escherichia coli, VSE, Acinetobacter spp.	
2.VRE, Staphylococcus Epidermidis, Staphylococcus Saprophyticus	
3.VSE, Acinetobacter spp., Candida spp.	
4.MSSA, Escherichia coli, Candida spp.	
5.MRSA, Corynebacterium	
6.Acinetobacter spp., Candida spp.	

MSSA: Methicillin sensitive staphylococcus aureus, MRSA: Methicillin resistant staphylococcus aureus, VSE: Vancomycin sensitive enterococcus, VRE: Vancomycin resistant enterococcus.

When comparing the bacteremia and non-bacteremia groups, a significant association was seen between BSI and clinical manifestation, as 10 out of 19 BSI patients had septic shock (p<0.001). Similarly, SCORTEN score was significantly associated with BSI as 8 out of 19 BSI patients had SCORTEN score of equal to or more than 4 (p=0.04). When comparing infective markers between the two groups, median procalcitonin was significantly raised in BSI group as compared to the non-bacteremia group (9.7ng/ml vs 0.4ng/ml – p=0.007). Regarding morbidity and mortality, all complications except acute kidney injury had a significant association with BSI as can be seen in Table-I. Similarly, admission to ICU was also seen to be more significantly associated with BSI (7 vs. 5 – p<0.001). Finally, out of a total 15 expiries, nine had BSI, showing a significant association between outcome and BSI (p<0.001). No significant association was seen between BSI and age, gender, disease classification, positive history of precipitating drug use, and length of stay.

## DISCUSSION

This study found BSI present in 19% of SJS, TEN, and overlap patients and identifies initial presentation of septic shock and a high SCORTEN score to be a predictor of developing BSI. The study establishes a significant association between BSI and development of complications including ICU admission leading to overall increased morbidity and mortality. Our study had a slightly higher percentage of population from the age group of more than 40 years. However, no significance determined between BSI and age > 40 years. A study from Asia indicated that people with age > 60 years who are affected by adverse drug reactions have a higher incidence of mortality and poor outcomes due to the frailty and presence of comorbidities.^16^ Similarly, Hirschmann JV et al.^8^ reported age greater than 40 years to be a significant factor for development of BSI. The lack of significance in our population might be due to the equal number of patients in both above and below 40 years of age. Whereas, a study performed at Kenyatta National Hospital reported 21-40 years of age as the most common age group of presentation.^17^

Our study showed female predominance (57%) towards SJS/TEN, which is comparable to studies conducted by Koh et al (59%) and de Prost et al (52.5%).^6^,^7^ Studies suggest having female sex is a risk factor for SCARs, however, no reasoning has been clearly identified in the literature.^1^ We found ischemic heart disease and respiratory diseases to have a significant association with incidence of BSI. Similarly, another study identified cardiovascular disease as an important risk factor for BSI at admission in these patients.^6^ However, de Prost et al concluded diabetes as an admission risk factor for BSI in SJS and TEN.

In our cohort *Staphylococcus aureus* (31.5%) and *Enterococcus* (26.3%) were the most common pathogens isolated in blood cultures. Almost all studies reported *Staphylococcus aureus* to be the most prevalent organism. de Prost et al and Lecadet A et al. demonstrated *Staphylococcus aureus* and *Pseudomonas aeruginosa* as the most common pathogens in their patients with SJS.^7^,^9^ Another study^6^ however, reported *Acinetobacter baumannii* (27.7%) as the most prevalent with *Staphylococcus aureus* (21.4%) following in second. We encountered polymicrobial BSIs in 6 (31.5%) patients, with one patient showing growth of four organisms i.e., *Staphylococcus aureus, E. coli, Acinetobacter*, and *Enterococcus* in a single blood culture. BSI in SJS and TEN patients tend to occur in polymicrobial combinations as reported by de Prost et al.^7^ in 24 (13.4%) and Koh et al.^6^ in 11.4% patients, while Hirschmann et al.^8^ reporting it to occur in almost half of the bacteremia cultures. Bacteremia due to *Enterobacteriaceae* was seen in only 4 blood cultures in our study which could be related to gut translocation and altered mucosal barrier of the gastrointestinal tract in patients with TEN as postulated by Early et al.^10^

Our study found procalcitonin to be a significant indicator for the development of BSI. Literature findings vary with some reporting no significant association between laboratory markers and BSI^7^, while other showing significant association between low hemoglobin and high CRP with occurrence of BSI.^6^ While our study did not look at other clinical markers like fever separately, we did find that a high SCORTEN score (> or equal to 4) carries a greater risk of developing BSI, leading to higher mortality. George et al reported a mortality rate of more than 70% at a SCORTEN score of 4 or more, in a study of 178 patients with acute skin failure admitted to ICU.^18^ However, a systematic review done on SJS/TEN patients using SCORTEN score revealed that the numbers of actual deaths were slightly lower than those projected by SCORTEN score regardless of the therapeutic intervention.^19^

Ahmed YI et al. reported 10 years (1990-2000) data of SJS/TEN including 101 patients from our institute.^1^ They concluded electrolyte imbalance and congestive heart failure as the most frequent complication after sepsis, whereas our data revealed electrolyte imbalance and AKI as the most common morbidity other than sepsis. Worth mentioning, we also assessed complications that were peculiar to patients with BSI in which deranged electrolytes (p<0.001), deranged coagulation (p<0.001) and respiratory failure/ICU admission (p<0.001) were significant. A retrospective analysis from the Chinese population showed AKI in only 14.9% of patients as an acute complication of SJS,^20^ which in contrast was seen in 43% of our patients.

Our patients also showed antibiotics as notable association with SJS/TEN as seen in studies from Ahmed YI et al. and Lerch M et al. However, our study found azithromycin to be the most common instigating agent, while in sharp contrast sulfonamides and penicillin group were the most common antibiotics to cause SJS/TEN in both studies.^1^,^21^ However, various review articles and case reports have reported SJS/TEN with the use of macrolides.^22^-^25^ A systematic review showed fluoroquinolones as the most frequently reported drug, whereas it was seen in only 3 patients from our study.^26^

In our study, the overall mortality was 15% while it was 60% in those who had BSI. In terms of ICU admissions, 12 patients with SJS/TEN were admitted to ICU out of which 5 (41%) died. de Prost et al. had reported a comparable overall mortality of 13.4% in their cohort of 179 patients, with 45.8% deaths in those who had BSI which is nearly similar to our findings.^7^ Though, Lecadet et al reported lower mortality (26%) in overall patients who had BSI.^9^ ICU admissions and related mortality were similar a 21 patients (48%) died.^9^ Literature suggests that patients with dermatological disorders including cellulitis, exfoliative dermatitis, autoimmune blistering dermatoses and cutaneous malignancies, complicated with BSI have prolonged intensive care stay as compared to general adult intensive care population.^18^

### Limitations:

Our study findings are unfortunately limited since it is a single-centered retrospective study with a small sample size. Furthermore, we did not include data regarding the treatment regimen offered to our patients in this study. However, data regarding SJS and TEN is scarce and we have reported a largest sample size of 100 patients from our population and region. Extensive studies are warranted to further determine and highlight which risk factors are important at admission and during the course of hospital stay for the occurrence of BSI.

## CONCLUSION

SJS/TEN is a life-threatening condition that can frequently be complicated by BSI. Our findings underscore the importance of vigilant monitoring and early detection of BSI in SJS/TEN patients, especially those presenting with high SCORTEN scores, to enhance clinical outcomes. Larger prospective studies with a control group are required to establish preventable risk factors during the hospital stay to prognosticate outcomes. Mitigating drug-related cutaneous adverse reactions requires comprehensive patient assessment and pharmacogenetic screening to identify high-risk individuals. Clinicians must consider cross-reactivity within drug classes and exercise caution in prescribing practices. Patient education on early signs of cutaneous adverse reactions is essential for timely intervention, potentially preventing progression to more severe outcomes.

### Authors Contribution:

**TA and ST** have made a substantial contribution to the conception and design of the manuscript.

**UJ** participated in data acquisition while **SBZM** in the analysis and interpretation.

All authors have participated in the drafting of the manuscript.

**SAA and MZM** revised it critically.

**TA** is responsible for the accuracy and integrity of the work.

All authors read and approved the final version of the manuscript.
